# Diseases of the Heart

**Published:** 1919-12

**Authors:** 


**Diseases of the Heart.** By FREDERICK W. PRICE, M.D. Pp. xiv., 472. London: Oxford Medical Publications. 1918. Price 21s. net.

Dr. F. W. Price is well known as an enthusiastic collector of polygraphic and electro-cardiographic records. Around an excellent selection of these he has put together a book which may be regarded as a very fair summary of "cardiology" viewed from the standpoint of the physiological school. The new syndromes of disordered function are clearly described, and the book should help medical men to understand what is admittedly a difficult bit of clinical medicine. The writer's standpoint may perhaps be gauged by the fact that he devotes fifteen pages to heart block and one to cardiac syphilis. We note that "mitral regurgitation" is still described as a disease. The new cardiology has not yet realised that it is only a symptom.

